# Beyond Taxonomy: Fungal Nutraceutical Supplementation Modifies the Functional Potential of the Gut Microbiome in Colorectal Cancer

**DOI:** 10.3390/ijms27198936

**Published:** 2026-10-08

**Authors:** Nahir Rodríguez-Costas, Arturo Rodríguez-Blanco, Cristina Regueiro Expósito, Joaquín Cubiella, Esteban Sinde Stompel, Catalina Fernández-de-Ana Portela, Astrid Irene Diez Martín, Pablo Taboada-Rodríguez, Belén Fontán Silva, María Dolores Rodríguez Fernández, Sara Zarraquiños, David Remedios, Cristina Alejandra Sánchez Gómez, Sara Alonso Lorenzo, Romina Fernández Poceiro, María Luisa de Castro Parga, Vicent Hernández Ramírez, Sonia Pérez-Castro

**Affiliations:** 1 Microbiology and Infectology Research Group, Galicia Sur Health Research Institute (IIS Galicia Sur), 36312 Vigo, Spain; 2 Functional Biology and Health Sciences Department, Universidade de Vigo, 36310 Vigo, Spain; 3 Hifas da Terra S.L., 36154 Pontevedra, Spain; arturo.rodriguez@hifasdaterra.com (A.R.-B.); esteban.sinde@hifasdaterra.com (E.S.S.); c.fdap@hifasdaterra.com (C.F.-d.-A.P.); 4 Research Group in Gastrointestinal Oncology Disease in Ourense, 32005 Ourense, Spain; cristina.regueiro@iisgaliciasur.es (C.R.E.); joaquin.cubiella.fernandez@sergas.es (J.C.); astrid.diez@iisgaliciasur.es (A.I.D.M.); sara.alonso.lorenzo@sergas.es (S.A.L.); romina.fernandez.poceiro@sergas.es (R.F.P.); maria.luisa.de.castro.parga@sergas.es (M.L.d.C.P.); vicent.hernandez.ramirez@sergas.es (V.H.R.); 5 Department of Gastroenterology, Hospital Universitario de Ourense, Centro de Investigación Biomédica en Red de Enfermedades Hepáticas y Digestivas (CIBEREHD), 32005 Ourense, Spain; sara.zarraquinos.martinez@sergas.es (S.Z.); david.rafael.remedios.espino@sergas.es (D.R.); cristina.alejandra.sanchez.gomez@sergas.es (C.A.S.G.); 6 Escuela Técnica Superior de Ingeniería (ETSE), Universidade de Santiago de Compostela, 15782 Santiago de Compostela, Spain; pablo.taboada@gmail.com; 7 Microbiology Department, Complexo Hospitalario Universitario de Vigo (CHUVI), 36312 Vigo, Spain; belen.fontan.silva@sergas.es (B.F.S.); maria.dolores.rodriguez.fernandez@sergas.es (M.D.R.F.); 8 Department of Gastroenterology, Complexo Hospitalario Universitario de Vigo, 36312 Vigo, Spain

**Keywords:** colorectal cancer, gut microbiome, fungal nutraceuticals, functional metagenomics, inflammation

## Abstract

The gut microbiome is increasingly recognized as an important determinant of host immune regulation, intestinal homeostasis, and recovery after colorectal cancer (CRC) surgery. Although our previous randomized, double-blind, placebo-controlled clinical trial demonstrated that fungal nutraceutical supplementation with Micodigest^®^ was associated with favorable taxonomic and systemic inflammatory changes in patients undergoing CRC surgery with curative intent, the functional consequences of this intervention remain poorly understood. Here, we performed a functional metagenomic analysis of fecal samples collected from patients enrolled in the original clinical trial to investigate whether Micodigest^®^ supplementation was associated with changes in the functional potential of the gut microbiome. A total of 37 patients were analyzed, including 24 receiving Micodigest^®^ and 13 receiving placebo, with paired samples collected before supplementation and after 4–6 weeks of intervention. Shallow shotgun metagenomic sequencing was performed, and microbial functional potential was characterized using KEGG Orthologues (KOs). Differential abundance analysis identified 14 significantly different KOs between groups at baseline and 23 after intervention, with no overlap between the two sets of orthologues. Following supplementation, Micodigest^®^-treated patients exhibited increased representation of functions related to CRISPR-associated systems, bacteriophage biology, and chitin-derived carbohydrate utilization, together with reduced representation of nutrient acquisition systems, conjugative transfer, sulfur metabolism, and components of the Wood–Ljungdahl pathway. These functional signatures suggest coordinated changes in microbial ecological adaptation, nutrient utilization, genetic exchange, and metabolic activity following fungal nutraceutical supplementation. Integration with previously reported taxonomic and inflammatory findings supports the hypothesis that Micodigest^®^ may promote coordinated remodeling of the gut microbiome and host–microbiota interactions during the perioperative period. Although the present study cannot establish causality or directly infer microbial activity from metagenomic potential, these findings provide novel functional insights into the microbiome response to fungal nutraceutical supplementation in patients with CRC and highlight functional metagenomics as a valuable complement to taxonomic profiling.

## 1. Introduction

Colorectal cancer (CRC) remains one of the leading causes of cancer-related morbidity and mortality worldwide, accounting for more than 1.9 million new cases and nearly a million deaths annually [1]. Despite major advances in screening programs, surgical techniques, presurgical management, and systemic therapies, curative-intent surgery continues to represent the cornerstone of treatment for localized disease [2].

Over the last decade, increasing evidence has demonstrated that the intestinal microbiota plays a critical role in colorectal carcinogenesis, host immune regulation, intestinal barrier integrity, and postoperative recovery. Alterations in gut microbial composition have been associated with tumor development, systemic inflammation, anastomotic leakage, surgical site infections, and other postoperative complications [3,4,5]. Experimental and clinical studies suggest that the microbiota may influence surgical outcomes through multiple mechanisms, including modulation of inflammatory responses, regulation of epithelial barrier function, production of bioactive metabolites, resistance to pathogen colonization and interactions with the host immune system [3,4,5].

These observations have led to an increasing interest in microbiota-targeted interventions as potential strategies to improve perioperative outcomes in patients with CRC. Among such interventions, fungal-derived nutraceuticals have attracted attention due to their immunomodulatory, antioxidant, anti-inflammatory, and microbiota-modulating properties [6]. Several bioactive compounds derived from edible and medicinal mushrooms, including β-glucans, terpenoids, and phenolic compounds, have been shown to influence both gut microbiome and host immune responses [7]. In this context, our group recently conducted a randomized, double-blind, placebo-controlled clinical trial that evaluated the effects of Micodigest^®^, a fungal nutraceutical formulation, in CRC patients undergoing surgery with curative intent. The study demonstrated that Micodigest^®^ supplementation was associated with favorable changes in inflammatory biomarkers, postoperative outcomes, and selected taxonomic features of the gut microbiota, supporting the hypothesis that modulation of the intestinal ecosystem may contribute to improved perioperative recovery [8]. Beyond taxonomic changes, the original trial also identified evidence of systemic immune modulation, particularly in patients undergoing conventional surgery, including significantly increased circulating lymphocyte counts together with a reduced neutrophil-to-lymphocyte ratio. These findings, together with the observed microbiota modifications, suggested that Micodigest^®^ supplementation may simultaneously influence microbial ecology and host immune responses, providing an important biological framework for the present functional metagenomic analysis.

Although taxonomic profiling has substantially improved our understanding of host-microbiota interactions, microbial community composition alone provides limited information regarding the functional capabilities of microbial communities. Distinct microbial taxa may perform similar biological functions through functional redundancy, whereas taxonomically similar communities may exhibit markedly different metabolic activities. Researching about the microbiome structure-function link is essential to better understand host-microbe interactions. Consequently, functional characterization has emerged as a critical component of microbiome research, particularly in complex clinical settings where microbial metabolic activity may be more relevant than taxonomic composition alone [9,10].

Shotgun metagenomic sequencing enables direct characterization of microbial functional potential through the identification of genes and metabolic pathways encoded within the microbiome [11]. One of the most widely adopted approaches for functional annotation is based on the Kyoto Encyclopedia of Genes and Genomes (KEGG), which organizes genes into evolutionarily conserved orthologous groups known as KEGG Orthologues (KOs) [12]. These orthologues represent functional units shared across different microbial taxa and provide a robust framework for investigating biological processes, metabolic pathways, and ecological functions within microbial communities [12]. Functional analyses based on KEGG orthologues have increasingly been used to identify microbial signatures associated with CRC, treatment response, inflammatory states, and clinical outcomes [13].

Functional metagenomics therefore offers the opportunity to identify biological mechanisms that may remain undetected by taxonomic analyses alone. Despite growing interest in microbiome-based interventions, the functional effects of fungal nutraceutical supplementation on the gut microbiome of patients with CRC remain largely unexplored. In particular, little is known regarding how such interventions influence microbial metabolic pathways, ecological resilience, host–microbiota interactions, or functional signatures associated with postoperative complications.

Therefore, the aim of the present study was to perform a comprehensive functional metagenomic characterization of fecal microbiota from patients enrolled in our previously published randomized clinical trial. Using shallow shotgun metagenomic sequencing and KEGG orthologue-based analyses, we investigated functional differences between Micodigest^®^- and placebo-treated patients according to surgical approach. By extending the findings of our previous taxonomic and clinical analyses, this study provides novel insights into the functional mechanisms through which microbiota modulation may influence perioperative outcomes in CRC patients.

## 2. Results

### 2.1. Study Cohort and Analytical Workflow

A total of 37 patients included in the previously published randomized clinical trial were analysed in the present functional metagenomic study. 24 patients received Micodigest^®^ and 13 received placebo. Fecal samples were collected before intervention (T1) and after completion of supplementation (T2), yielding a total of 74 samples available for analysis. The study cohort included 10 patients who underwent robotic surgery and 27 who underwent conventional surgery.

Detailed demographic, clinical, inflammatory, and taxonomic characteristics of the cohort have been reported previously [8]. As previously reported, Micodigest^®^ supplementation was associated with a significant increase in circulating lymphocyte counts together with a significant reduction in the neutrophil-to-lymphocyte ratio in patients undergoing conventional surgery, supporting a systemic immunomodulatory effect of the intervention.

The primary objective of the present work was to identify functional differences in the gut microbiome associated with Micodigest^®^ supplementation through differential abundance analysis of KEGG orthologues (KOs). The functional differences identified at baseline (T1) and after supplementation (T2) are presented in the following sections, together with the corresponding pathway-level analyses. In addition, the functional signatures identified were evaluated in the context of the taxonomic alterations previously reported in the original study to explore potential biological relationships between microbial composition and microbial functional potential. Exploratory analyses stratified according to surgical approach were also performed and are presented in the Supplementary Material.

### 2.2. Baseline Functional Differences Between Micodigest^®^ and Placebo Groups

Differential abundance analysis performed on baseline samples (T1) identified 14 significantly different KEGG orthologues between patients assigned to the Micodigest^®^ and placebo groups. As the study did not include a healthy control group, these baseline differences cannot be used to define functional features specifically associated with colorectal cancer or to characterize tumour-associated dysbiosis relative to a healthy microbiome.

The differentially abundant orthologues were distributed across several functional categories, including transport systems, metabolic enzymes, transcriptional regulators, and DNA-associated proteins. No complete KEGG pathway was represented by multiple significantly altered orthologues. None of the 14 differentially abundant orthologues identified at T1 overlapped with the orthologues identified in the post-intervention analysis at T2, indicating that the functional signatures detected at the two time points were distinct. The complete list of differentially abundant orthologues identified at baseline is shown in Supplementary Table S1.

### 2.3. Functional Differences Between Micodigest^®^ and Placebo After Intervention

Analysis of post-intervention samples (T2) identified 64% more significantly different KEGG orthologues (23 differentially abundant KEGG orthologues) between Micodigest^®^- and placebo-treated patients, as compared to baseline. Interestingly, these 23 orthologues did not match the 14 found in T1.

Among the orthologues over-represented in the Micodigest^®^ group were functions associated with CRISPR-related systems and bacteriophage-associated proteins, sulfur metabolism, including *csx10* and bacteriophage DNA polymerase. Conversely, *cysP*, *acsB*, and *acsD* which are associated with sulfate/thiosulfate transport and carbon fixation pathways, were underrepresented in the Micodigest^®^ group (Table 1).

**Figure 1:**
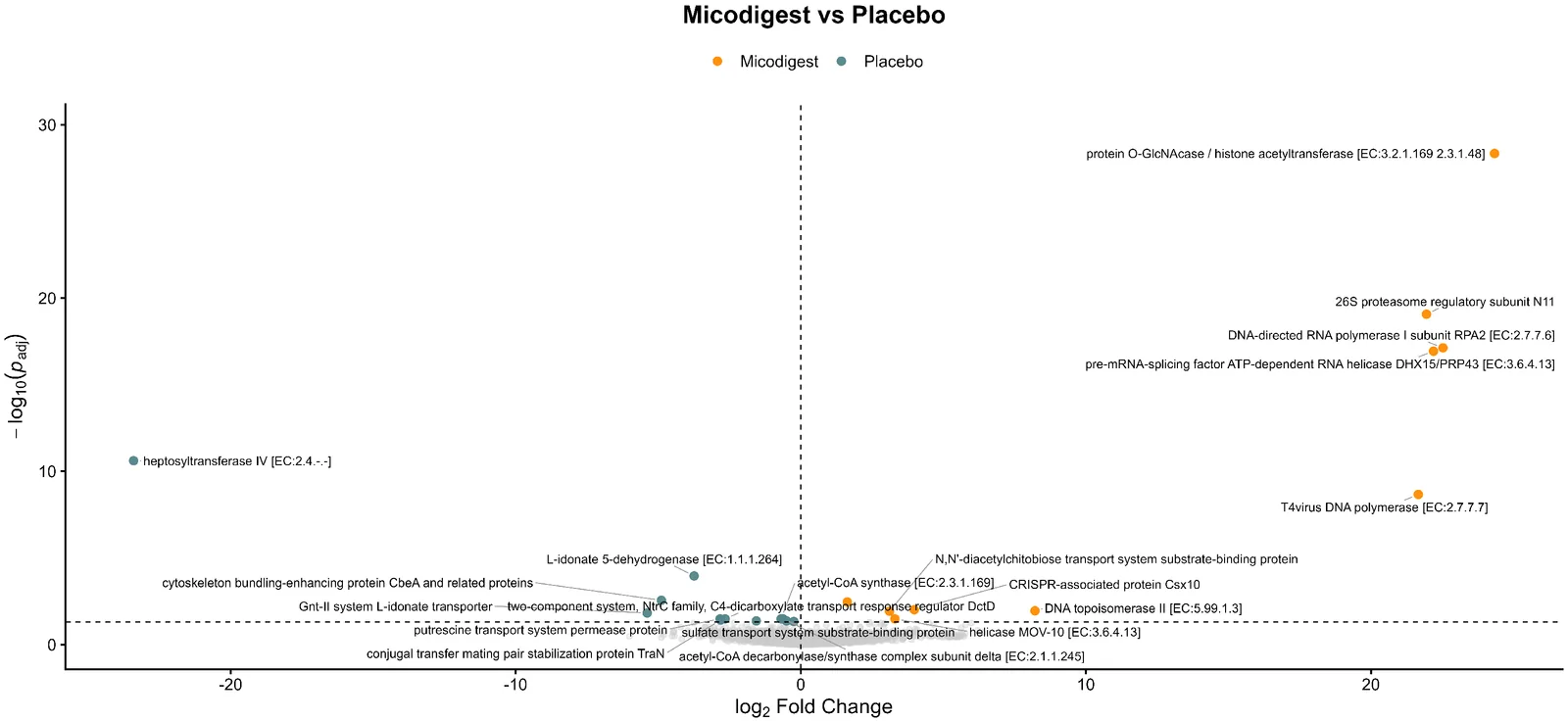
Volcano plot of differentially abundant orthologues identified at T2. The orange dots on the right side of the graph correspond to genes significantly over-represented in the Micodigest^®^-treated group, and the blue dots on the left are genes significantly over-represented in the placebo group.

Conversely, orthologues over-represented in placebo-treated patients included functions related to nutrient transport systems, regulatory proteins, and conjugative transfer mechanisms; including *potI*, *msmG*, *dctD*, and *traN* (Table 1). The complete list of significantly different orthologues is presented in Figure 1.

**Figure 2:**
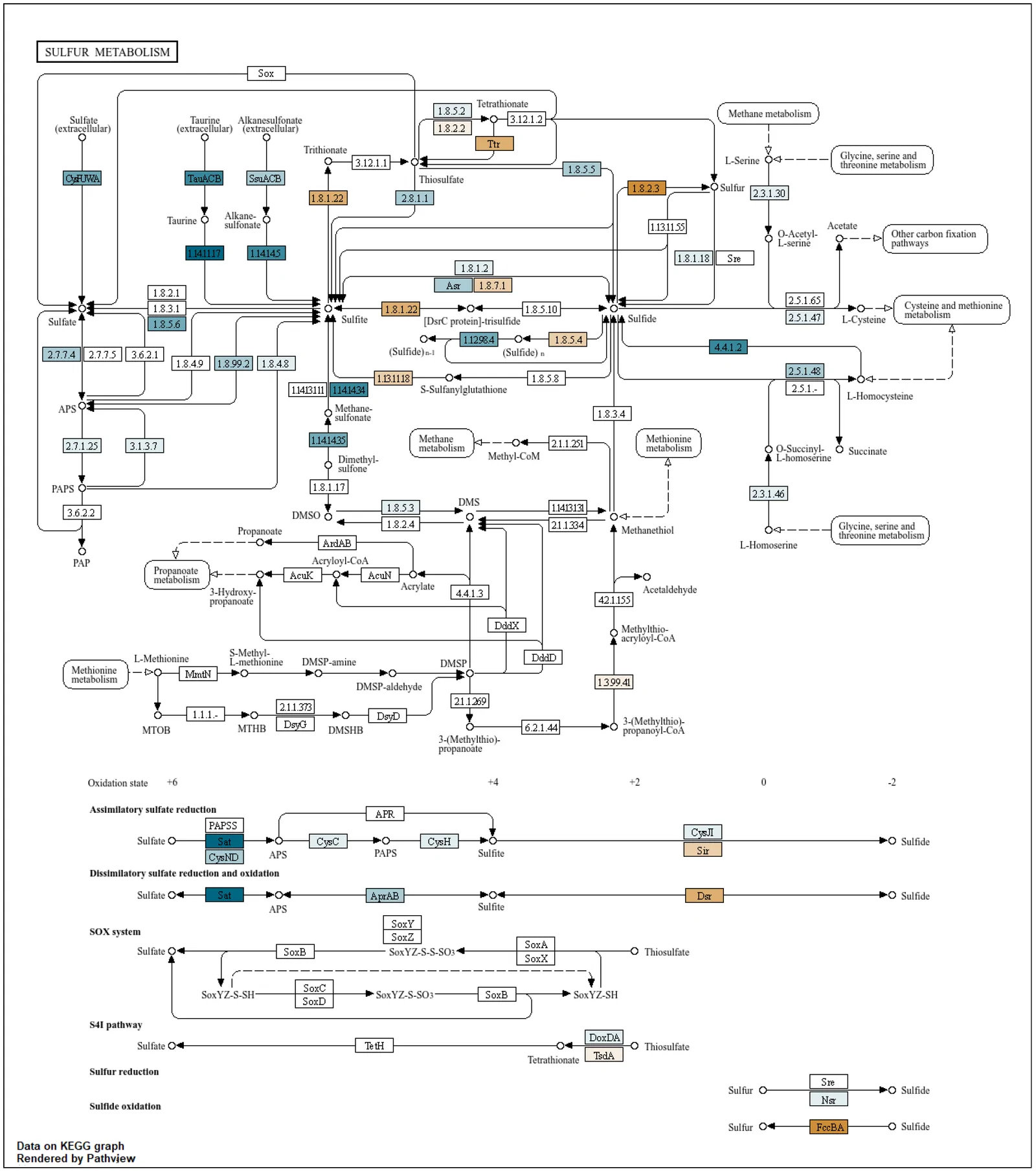
Sulfur metabolism pathway (map00920). Positive $Log_{2}FoldChange$ values (orange) correspond to over-represented orthologs in Micodigest^®^ at T2, and negative values (blue) to under-represented orthologs in Micodigest^®^ at T2. Enzyme significantly under-represented in Micodigest^®^ (*CysPUWA*) is shown in blue ($p_{Adj}$ = 3.27 × $10^{-2}$).

**Figure 3:**
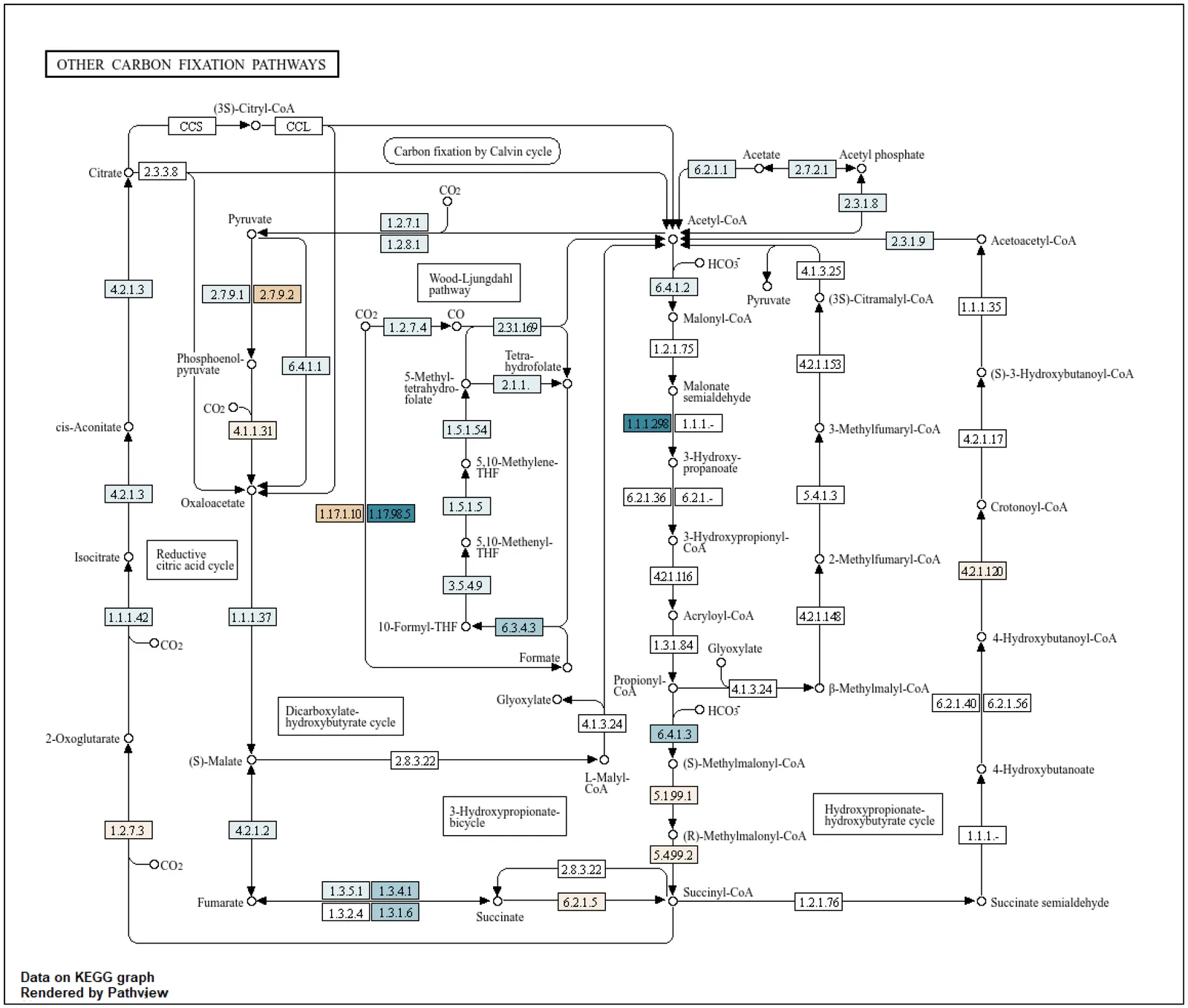
Other carbon fixation pathways (map00720). Positive $Log_{2}FoldChange$ values (orange) correspond to over-represented orthologs in Micodigest^®^ at T2, and negative values (blue) to under-represented orthologs in Micodigest^®^ at T2. Enzymes significantly under-represented in Micodigest^®^ (*AcsB* and *CdhD*; 2.3.1.169 both genes) are shown in blue ($p_{Adj}$ = 3.27 × $10^{-2}$ and $p_{Adj}$ = 3.27 × $10^{-2}$, respectively).

Several differentially abundant orthologues identified at T2 mapped to common KEGG pathways and were therefore selected for pathway-level visualization. Two representative KEGG pathways were selected because they contained multiple significantly altered orthologues. Sulfur metabolism was selected due to the differential abundance of the sulfate/thiosulfate transport protein *cysP* (Figure 2), whereas the Wood–Ljungdahl pathway was selected because two components of the acetyl-CoA synthase/carbon monoxide dehydrogenase complex, *acsB* and *acsD*, were simultaneously differentially abundant between groups (Figure 3). All three orthologues were under-represented in the Micodigest® group at T2, indicating a coordinated reduction in the representation of functions involved in sulfate/thiosulfate uptake and the acetyl-CoA synthase/carbon monoxide dehydrogenase complex. The ABC transporters pathway, which includes several of the orthologs mentioned, is shown in Supplementary Figure S1.

Pathway mapping highlighted sulfur utilization and Wood–Ljungdahl-associated carbon fixation as two functional processes showing coordinated differences between treatment groups, providing a pathway-level representation of the metagenomic changes observed after supplementation.

### 2.4. Exploratory Analyses According to Surgical Approach

Additional differential abundance analyses were performed separately in robotic and conventional surgery subgroups to explore whether the functional response to Micodigest^®^ differed according to surgical approach.

No differentially abundant KEGG orthologues were identified between the Micodigest^®^ and placebo groups within the robotic surgery subgroup (N = 10). In contrast, 14 differentially abundant KEGG orthologues were identified within the conventional surgery subgroup (N = 27) (Supplementary Table S2). Given the small sample size of the robotic surgery subgroup and the exploratory nature of this analysis, these findings should be interpreted cautiously and do not allow conclusions regarding an independent effect of surgical approach on microbiome functional composition.

## 3. Discussion

The present study provides the first functional metagenomic characterization of the gut microbiome in CRC patients receiving Micodigest^®^ supplementation during the presurgery period. Based on our previously reported taxonomic findings, we investigated whether the microbial compositional changes induced by this fungal nutraceutical were accompanied by alterations in the microbial functional potential. Several relevant observations emerged from the analysis.

First, functional differences between Micodigest^®^- and placebo-treated patients increased after supplementation, with the number of significantly altered KEGG orthologues rising from 14 at baseline to 23 following intervention. Notably, the 23 orthologues identified at T2 did not overlap with those detected at baseline, suggesting that the post-intervention functional profile was not merely a persistence of pre-existing differences between groups but rather reflected newly emerging functional signatures after supplementation. Because baseline samples were collected before treatment initiation, the larger number of functional differences observed at T2 suggests a potential functional remodeling of the gut microbiome associated with Micodigest^®^ supplementation during the perioperative period. These findings extend our previous taxonomic observations and indicate that fungal-derived nutraceuticals may influence not only microbial composition but also the biological activities encoded within the intestinal microbial community.

This distinction is particularly relevant because microbial functions often represent a more direct proxy of host-microbiome interactions than taxonomy alone. Functional redundancy allows different microbial taxa to perform similar metabolic activities, whereas taxonomically similar communities may display substantially different functional capacities [10]. Consequently, functional metagenomics has increasingly emerged as a complementary approach to taxonomic profiling for understanding microbiome-mediated effects in human disease [14].

### 3.1. Micodigest^®^ Supplementation Is Associated with Functional Remodeling of Nutrient Utilization, Microbial Ecological Dynamics and Community Adaptation

The present study revealed that Micodigest^®^ supplementation was associated with distinct functional changes in the gut microbiome, particularly involving orthologues related to microbial ecological interactions, nutrient acquisition, sulfur metabolism, and carbon utilization [3]. Alterations in these processes have been linked to intestinal inflammation, epithelial dysfunction, microbial resilience, and tumor-associated dysbiosis [4]. Notably, these differences became evident after supplementation, supporting the hypothesis that fungal-derived bioactive compounds may contribute to a potential functional remodeling of the intestinal microbiome.

Among the orthologues over-represented in Micodigest^®^-treated patients, the CRISPR-associated protein *Csx10* and the bacteriophage DNA polymerase *Gp43* were of particular interest. Although these orthologues belong to different biological systems, both participate in processes that influence microbial adaptation, genetic exchange, and ecosystem dynamics [15]. CRISPR-Cas systems constitute one of the main microbial defense mechanisms against invading genetic elements, including bacteriophages and mobile genetic elements. Beyond their canonical antiviral role, CRISPR systems are increasingly recognized as important regulators of microbiome stability, horizontal gene transfer, and microbial community resilience [16,17].

Similarly, bacteriophages have emerged as key ecological regulators of intestinal microbial ecosystems. Through predator–prey interactions, phages shape bacterial population structure, influence metabolic activity, and contribute to microbiome adaptation under environmental stress [18]. Recent evidence indicates that phage–bacteria interactions play important roles in maintaining gut ecosystem homeostasis and may influence inflammatory and metabolic processes relevant to CRC [19,20].

Interestingly, the original taxonomic analysis of this cohort demonstrated over-representation of several bacterial taxa potentially associated with intestinal health following Micodigest^®^ supplementation. Although direct assignment of individual orthologues to specific taxa was beyond the scope of the present analysis, the concurrent observation of taxonomic shifts together with increased representation of CRISPR- and phage-related functions suggests that Micodigest^®^ may influence not only microbial composition but also ecological processes governing adaptation, resilience, and microbial community restructuring within the intestinal ecosystem.

In addition to these ecological signatures, several orthologues associated with nutrient acquisition and environmental sensing were significantly down-represented in Micodigest^®^-treated patients. These included the L-idonate transporter *IdnT* and the L-idonate 5-dehydrogenase *IdnD*, which participate in the uptake and utilization of alternative carbohydrate substrates, as well as *PotI*, a component of the putrescine transport system, *DctD*, a transcriptional regulator controlling C4-dicarboxylate utilization, and *MsmG*, a transporter involved in raffinose-family oligosaccharide uptake. Although these functions participate in distinct metabolic pathways, they share a common ecological role in facilitating the acquisition and utilization of environmental nutrients [21,22,23].

The coordinated reduction of these orthologues may therefore reflect a decreased reliance on specialized nutrient-scavenging strategies within the microbial community. In microbial ecosystems, increased representation of substrate-specific transport systems is frequently associated with competition for limited resources and adaptation to fluctuating nutrient availability [24,25]. Conversely, reduced dependence on such systems may indicate greater metabolic integration, cross-feeding interactions, and more efficient utilization of available substrates within the community [26]. While ecological stability was not directly assessed in the present study, the overall pattern observed after Micodigest^®^ supplementation is compatible with a potential shift toward a functionally more balanced microbial ecosystem characterized by reduced selective pressure for competitive nutrient acquisition.

One possible explanation for this observation is the complex composition of Micodigest^®^, which combines extracts from multiple mushroom species with fructooligosaccharides/inulin and other nutritional compounds. In particular, mushroom-derived polysaccharides, including β-glucan-rich fractions, may provide fermentable substrates for specific microbial populations or otherwise interact with the intestinal microbiota, thereby modifying microbial substrate availability and host–microbiome interactions. The fructooligosaccharides/inulin present in the formulation may also contribute to these effects by influencing microbial substrate availability and community composition. Although these mechanisms are biologically plausible and require experimental confirmation, they are consistent with evidence supporting prebiotic-like effects of mushroom-derived polysaccharides on gut microbial communities [27]. However, the present study was not designed to determine which specific component or bioactive compound is responsible for the observed functional changes.

Additional orthologues related to microbial adaptation and community interactions further support this interpretation. The conjugative transfer protein *TraN* was significantly under-represented in Micodigest^®^-treated patients. Unlike CRISPR-associated proteins and bacteriophage-related functions, *TraN* participates in bacterial conjugation and horizontal gene transfer through plasmid-mediated mechanisms [28]. Reduced abundance of this function may therefore indicate altered dynamics of mobile genetic elements and bacterial genetic exchange within the intestinal ecosystem. Together with the over-representation of CRISPR- and phage-associated orthologues, these findings suggest that Micodigest^®^ supplementation may influence multiple layers of microbial ecological regulation, including defense systems, viral interactions, and horizontal gene transfer processes.

In contrast, two relevant orthologues were over-represented following Micodigest^®^ supplementation. *DasA*, a substrate-binding component of the N,N′-diacetylchitobiose transport system, participates in the uptake of chitin-derived oligosaccharides and structurally related carbohydrates [29]. Given that fungal cell walls are rich in chitin-associated compounds [30], over-representation of *DasA* may reflect adaptation of specific microbial populations capable of utilizing substrates derived from fungal bioactive ingredients. Likewise, *WbyK*, a mannosyltransferase involved in glycan biosynthesis and cell-envelope assembly, was also over-represented after supplementation. Functions associated with bacterial surface glycosylation contribute to microbial adhesion, cell-envelope architecture, inter-bacterial interactions, and host–microbiota communication [31,32], suggesting that Micodigest^®^-associated functional changes may extend beyond metabolism to include microbial surface remodeling and ecological adaptation.

Furthermore, the remaining significant orthologues included functions related to sulfur metabolism and the Wood–Ljungdahl carbon fixation pathway, providing two coherent pathway-level functional signatures associated with Micodigest^®^ supplementation. Importantly, these pathways were not interpreted as significantly altered complete metabolic pathways; rather, they were highlighted because multiple differentially abundant orthologues mapped to biologically related processes, providing a pathway-level context for the functional differences observed between treatment groups.

The sulfur metabolism signature was represented primarily by *CysP*, a substrate-binding protein of the sulfate/thiosulfate transport system, which was significantly under-represented in Micodigest^®^-treated patients at T2. Sulfate and thiosulfate availability can influence microbial sulfur metabolism and the activity of sulfur-transforming microorganisms. In the intestinal environment, microbial sulfur metabolism is of particular interest in CRC because sulfur-containing substrates can contribute to the production of hydrogen sulfide ($H_{2}$S). Although, $H_{2}$S has physiological functions at low concentrations, excessive microbial $H_{2}$S production has been associated with epithelial barrier disruption, mucosal injury, DNA damage, and inflammatory processes relevant to colorectal carcinogenesis [33,34]. Several studies have demonstrated increased abundance of sulfur-metabolizing microorganisms in CRC and inflammatory intestinal conditions [34,35]. Therefore, the reduced representation of *CysP* observed after Micodigest^®^ supplementation may indicate a decreased metagenomic potential for sulfate/thiosulfate acquisition. However, because *CysP* represents a transport function rather than a direct measurement of sulfur-reducing activity or $H_{2}$S production, this finding should not be interpreted as evidence of reduced intestinal $H_{2}$S levels. Rather, it identifies sulfur substrate acquisition as one of the functional processes potentially modified following supplementation.

The second functional signature involved the Wood–Ljungdahl pathway and related carbon fixation functions, represented by the reduced abundance of the acetyl-CoA synthase complex components *AcsB* and *AcsD*. The Wood–Ljungdahl pathway constitutes a major microbial carbon fixation route used by acetogenic and anaerobic microorganisms [36,37]. The simultaneous reduction of two components of the same acetyl-CoA synthase/carbon monoxide dehydrogenase complex is therefore noteworthy because it suggests that the observed difference was not restricted to a single orthologue, but involved multiple components of a functionally connected carbon metabolic system [38]. This pattern may reflect changes in the representation of microorganisms or microbial populations capable of acetogenic carbon fixation and anaerobic carbon metabolism following supplementation.

Nevertheless, the biological interpretation of this finding requires caution. Reduced representation of *AcsB* and *AcsD* does not necessarily indicate a generalized reduction in microbial carbon fixation, nor does it demonstrate altered acetogenesis or carbon flux in vivo. Rather, these metagenomic data indicate a change in the functional potential encoded by the microbial community. Together with the concurrent alteration in the sulfate/thiosulfate transport function *CysP*, the *AcsB/AcsD* findings suggest that Micodigest^®^-associated functional remodeling extended to microbial pathways involved in sulfur substrate acquisition and anaerobic carbon metabolism. These changes may reflect broader reorganization of microbial metabolic networks and resource utilization within the intestinal ecosystem, although metatranscriptomic, metabolomic, and metabolic flux measurements would be required to determine whether these changes translate into altered pathway activity or metabolite production.

Importantly, none of these functional signatures could have been inferred solely from taxonomic profiling. The combined observation of changes affecting microbial nutrient acquisition, ecological interactions, sulfur utilization, carbon metabolism, and horizontal gene transfer highlights the added value of functional metagenomics for understanding microbiome-mediated mechanisms associated with fungal nutraceutical supplementation in patients with CRC.

### 3.2. Integration with Previous Taxonomic Findings and Clinical Implications

The present functional analysis substantially complements and extends the taxonomic findings previously reported from this randomized clinical trial. While the original study demonstrated that Micodigest^®^ supplementation modified gut microbial composition and was associated with favorable inflammatory and clinical outcomes, the current metagenomic analysis indicates that these compositional changes were accompanied by coordinated alterations in microbial functional potential. Importantly, the functional signatures identified after supplementation were largely related to microbial ecological interactions, nutrient acquisition strategies, sulfur metabolism, carbon utilization and genetic exchange, suggesting that fungal-derived bioactive compounds may influence not only which microorganisms are present within the intestinal ecosystem but also how these communities function and adapt.

The integration of taxonomic and functional analyses provides a more comprehensive interpretation of the microbiome response to Micodigest^®^ supplementation. Functional redundancy within the gut microbiome means that similar biological activities may be maintained despite taxonomic variability, whereas relatively modest compositional changes may be accompanied by substantial functional reorganization. Consequently, the observation that supplementation was associated with coherent changes affecting multiple ecological processes (including CRISPR-associated defense systems, bacteriophage-related functions, nutrient transport, sulfur utilization and carbon metabolism) suggests that Micodigest^®^ may promote microbiome adaptation at several complementary biological levels rather than through isolated metabolic effects.

Although the present dataset does not allow definitive attribution of individual orthologues to specific microbial taxa, the functional signatures identified after supplementation are biologically consistent with mechanisms that have previously been associated with beneficial microbiome modulation, including enhanced ecological resilience, increased community adaptation, altered microbial nutrient utilization and reduced representation of functions linked to excessive sulfur metabolism. Together with our previously reported enrichment of bacterial taxa associated with intestinal health, these observations support the hypothesis that fungal supplementation promotes coordinated ecological restructuring of the gut microbiome.

The functional changes identified in the present study also provide a plausible mechanistic framework for the taxonomic alterations previously reported in this cohort. The original clinical trial demonstrated that Micodigest^®^ supplementation induced taxonomic remodelling of the gut microbiota, including a reduced relative abundance of *Firmicutes* and *Actinobacteria* together with changes affecting several bacterial families and genera [8]. Although individual orthologues cannot be directly assigned to specific microbial taxa, several of the functions identified here (including CRISPR-associated systems, bacteriophage-related orthologues, reduced nutrient-scavenging transporters and altered sulfur metabolism) are compatible with microbial communities undergoing ecological restructuring and adaptation. Thus, the present functional data extend our previous taxonomic observations by providing potential biological mechanisms underlying these compositional changes.

An additional aspect that reinforces the biological relevance of the present findings is their consistency with the systemic immune modulation previously observed in the same randomized clinical trial. Micodigest^®^ supplementation significantly increased circulating lymphocyte counts while reducing the neutrophil-to-lymphocyte ratio in patients undergoing conventional surgery [8]. The neutrophil-to-lymphocyte ratio is widely recognised as an integrative biomarker of systemic inflammation and immune competence, and its reduction has been associated with a transition from a predominantly pro-inflammatory state towards a more regulated immune environment [39,40], as well as with improved prognosis following colorectal surgery [41]. Although no individual orthologue could be directly linked to immune-cell regulation, several of the functional changes identified in the present study are consistent with microbial ecological states that have previously been associated with improved intestinal homeostasis and host immune regulation. Therefore, although our functional metagenomic analyses cannot establish causality, the convergence of microbial functional remodelling with independent systemic immune biomarkers strengthens the biological plausibility of a coordinated microbiota–host response induced by Micodigest^®^ supplementation.

However, the clinical significance of the observed functional changes should be interpreted with caution. The present data do not allow these alterations to be classified as uniformly beneficial or detrimental to patients, as functional metagenomics provides information on microbial functional potential rather than direct measurements of microbial activity, metabolite production, or host physiological effects. Although clinical and inflammatory outcomes from the original randomized clinical trial were available and have been reported previously, the present secondary analysis was not designed to establish direct associations between individual microbial functional features and patient outcomes. The concurrent observation of microbiome functional changes and systemic immune alterations therefore does not establish that the former contributed to the latter. Future studies integrating longitudinal functional metagenomics with clinical outcomes, metabolomic measurements, and host immune profiling will be required to determine whether specific microbial functional changes are associated with clinically meaningful effects.

Interestingly, these observations are also consistent with previous evidence regarding *Ganoderma lucidum*, one of the principal components of the Micodigest^®^ formulation. Previous clinical studies have shown that *G. lucidum* supplementation increases *CD3+*, *CD4+* and *CD8+* lymphocyte populations in patients with CRC, supporting its immunomodulatory properties [42]. The increase in circulating lymphocytes observed in our original trial therefore agrees with these previous findings and provides independent clinical support for the functional microbial changes identified in the present study, suggesting that fungal supplementation may simultaneously influence microbial ecology and host immune regulation.

Taken together, the taxonomic, functional and systemic immune findings obtained from the same randomized clinical trial appear remarkably coherent. Taxonomic analyses demonstrated restructuring of the intestinal microbiota, functional metagenomics identified coordinated alterations in microbial ecological and metabolic capabilities, while clinical analyses revealed evidence of systemic immune modulation through increased lymphocyte counts and reduced neutrophil-to-lymphocyte ratios. Although these observations do not demonstrate causal relationships, their convergence supports the hypothesis that Micodigest^®^ supplementation promotes coordinated host–microbiota interactions rather than isolated microbiological changes. Beyond providing mechanistic insight into the biological effects of Micodigest^®^ supplementation, the present findings also suggest that functional microbiome profiling may eventually contribute to perioperative risk stratification and patient selection for microbiome-targeted interventions. Future prospective studies integrating shotgun metagenomics with metatranscriptomics, metabolomics and host immune profiling will be required to determine whether these functional signatures represent causal mechanisms underlying postoperative recovery and whether they can be therapeutically modified before surgery.

Given the multifactorial composition of Micodigest^®^ and its potential interactions with the gut microbiota, future studies may investigate whether similar microbiome-related effects are observed in other cancer populations or in non-cancer-related conditions in which modulation of the intestinal microbiome may be relevant. However, the present findings are specific to the studied colorectal cancer population and should not be extrapolated to other clinical applications without dedicated clinical investigation.

### 3.3. Strengths and Limitations

Several strengths should be acknowledged. This study was performed within a randomized, double-blind, placebo-controlled clinical trial, minimizing potential confounding factors. The availability of paired longitudinal samples from the same patients at baseline and after completion of supplementation allowed evaluation of microbiome functional dynamics during the perioperative period. Furthermore, integration of functional metagenomics with previously published taxonomic analyses provided a substantially more comprehensive characterization of microbiome responses to Micodigest^®^ supplementation than either approach alone, enabling identification of functional signatures that could not have been inferred solely from microbial composition.

Nevertheless, certain limitations should also be considered. The relatively small cohort size limits statistical power and may have prevented detection of additional functionally relevant orthologues. Functional annotation was inferred from shotgun metagenomic sequencing and therefore reflects the functional potential encoded by the microbiome rather than actual gene expression or metabolic activity. Consequently, the biological consequences of the observed functional alterations remain inferential and should be confirmed by complementary metatranscriptomic, metaproteomic or metabolomic analyses. In particular, microbial metabolites such as short-chain fatty acids (SCFAs) were not measured in the present study. Therefore, the observed metagenomic changes cannot be directly linked to alterations in microbial metabolite production. Future studies combining shotgun metagenomics with targeted metabolomic analyses, including quantitative assessment of fecal SCFAs, would help determine whether the functional changes identified after Micodigest^®^ supplementation are accompanied by corresponding metabolic alterations. In addition, assignment of individual orthologues to specific bacterial taxa was not possible, precluding direct identification of the microorganisms responsible for the observed functional changes.

Importantly, the original clinical trial included fecal sampling only at baseline and after completion of the intervention; therefore, no intermediate samples were available to characterize the temporal trajectory of microbiome functional changes during supplementation. As a result, the present analysis cannot determine when the observed functional alterations emerged or whether they evolved progressively throughout the intervention period.

An additional limitation is that the original clinical trial did not include a healthy control group. Consequently, the present metagenomic analysis cannot distinguish functional features associated specifically with colorectal cancer from inter-individual or disease-related variation within the CRC cohort. The baseline analysis was therefore restricted to comparisons between the randomized Micodigest^®^ and placebo groups, while longitudinal changes were assessed between T1 and T2 within the available paired samples.

Additionally, although clinical and postoperative outcomes were available from the original clinical trial, the present secondary analysis was not designed to establish direct associations between specific microbial functional features and patient outcomes. Therefore, the clinical relevance of the observed functional alterations remains uncertain, and the present data do not allow these changes to be interpreted as uniformly beneficial or detrimental. Larger independent cohorts with more frequent longitudinal sampling and integrated microbiome, metabolomic, immune, and clinical assessments will be required to determine whether specific functional signatures are associated with clinically meaningful outcomes and whether these associations are reproducible.

Finally, cognitive and gut–brain axis-related outcomes were not assessed in the original clinical trial. Therefore, the present study cannot determine whether the microbiome functional changes observed after Micodigest^®^ supplementation were associated with cognitive, psychological, or other gut–brain axis-related effects. Future studies incorporating validated neurocognitive and patient-reported assessments together with microbiome and metabolomic analyses would be required to investigate this potential relationship.

## 4. Materials and Methods

### 4.1. Study Design and Patient Population

This study represents a secondary functional metagenomic analysis derived from the randomized, double-blind, placebo-controlled clinical trial previously conducted by our group to evaluate the effects of the fungal nutraceutical supplement Micodigest^®^ in patients undergoing CRC surgery with curative intent [8].

The original trial included patients diagnosed with colorectal adenocarcinoma who were randomly assigned to receive either Micodigest^®^ (N = 24) or placebo (N = 13) before surgery, according to the study protocol. Participants underwent either robotic-assisted surgery (N = 10) or conventional surgery (N = 27) (Figure 1). In this substudy, only paired samples were selected (patients who provided their sample in T1 and T2).

Micodigest^®^ 2.0 was provided by Hifas da Terra as a commercial fungal nutraceutical formulation. 30 capsules and 300 mL of syrup are supplied and contained water, organic agave nectar, γ-cyclodextrin, fructooligosaccharides containing 90% inulin (4.2%), *Cordyceps sinensis* extract (3.2%), organic *Ganoderma lucidum* extract (3.2%), organic *Lentinula edodes* extract (3.2%), organic *Polyporus umbellatus* extract (3.2%), organic *Agaricus blazei* extract (2.5%), phosphatidylserine (soy-containing), glycine as a flavour enhancer, organic *Grifola frondosa* extract (2.4%), organic *Hericium erinaceus* extract (2.4%), organic *Inonotus obliquus* extract (2.4%), organic acerola (*Malpighia glabra* L.) concentrated juice (1.7%), xylitol and sucralose, citric acid, L-ascorbic acid, strawberry and vanilla flavourings, potassium sorbate, nicotinamide, cholecalciferol, selenium methionine, calcium pantothenate, retinol, pyridoxine hydrochloride, thiamine hydrochloride, riboflavin, folic acid, sodium selenite, biotin, and cyanocobalamin. The nutritional contribution per 10.5 mL dose was vitamin A (200 µg), vitamin B1 (0.55 mg), vitamin B2 (0.7 mg), vitamin B3 (8 mg), vitamin B5 (3 mg), vitamin B6 (0.7 mg), vitamin B7 (25 µg), vitamin B9 (100 µg), vitamin B12 (1.25 µg), vitamin C (40 mg), vitamin D3 (20 µg), and selenium (45 µg). The formulation therefore contained extracts from multiple mushroom species rather than a single *G. lucidum* extract. The available product information specifies the relative proportion of the mushroom extracts but does not provide the quantitative concentrations of individual bioactive constituents within these extracts, such as β-glucans, triterpenoids, or other mushroom-derived metabolites. Consequently, the contribution of individual bioactive compounds cannot be quantitatively determined from the formulation information available for the present study. The placebo contained no fungal extracts and was matched in taste and texture and manufactured by the same company. Both products were stored at room temperature and protected from light. Detailed information regarding patient recruitment, eligibility criteria, randomization procedures, and clinical outcomes has been reported previously [8].

For the present study, fecal samples obtained from study participants were analysed using shallow shotgun metagenomic sequencing. Samples were collected at two predefined time points: baseline (T1), before probiotic or placebo supplementation and post-intervention (T2), after 4–6 weeks and previous to surgical treatment. No intermediate fecal samples were collected during the intervention period in the original clinical trial. Therefore, the present secondary analysis was limited to these two longitudinal time points.

**Figure 4:**
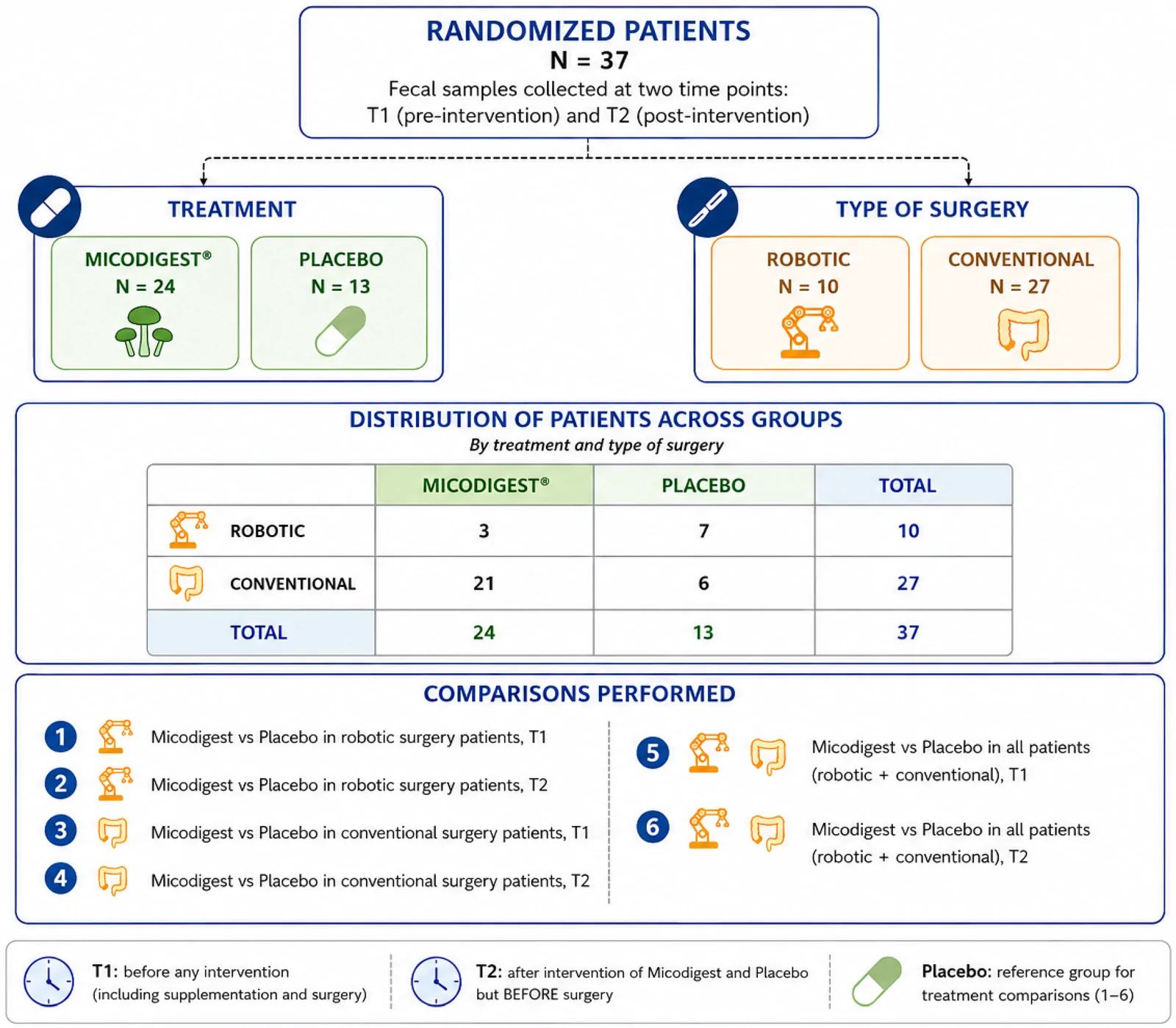
Experimental design of the study. Explanation of the different comparisons made in the subsequent statistical analyses along with the sample size of each group. This image has been enhanced with generative artificial intelligence.

Functional analyses were performed separately in patients undergoing robotic surgery (N = 10), conventional surgery (N = 27), and in the overall cohort combining both surgical approaches (N = 37) (Figure 4). This comparison was designed to explore microbial functional signatures associated with the reduction in the inflammatory parameters observed in the group treated with Micodigest^®^.

### 4.2. DNA Extraction, Shotgun Metagenomic Sequencing and Functional Annotation

DNA extraction, library preparation, and sequencing procedures were performed as previously described in the original clinical trial [8].

Briefly, total microbial DNA was extracted from fecal samples using validated protocols for human stool specimens. Sequencing libraries were prepared according to Illumina recommendations and sequenced using shallow shotgun metagenomics, generating a mean sequencing depth of approximately 5.5 million paired-end reads per sample.

Raw sequencing reads were processed using the fully automated *SqueezeMeta* pipeline (*v1.6.3*) (https://github.com/jtamames/SqueezeMeta, accessed on 13 August 2026) executed at the Galicia Supercomputing Centre (CESGA, Spain). Functional annotation was performed against the Kyoto Encyclopedia of Genes and Genomes (KEGG) database (https://www.genome.jp/kegg/, accessed on 9 August 2026), generating abundance matrices of KEGG Orthologues (KOs) for downstream analyses.

The resulting data were imported into R (*version 4.5.0*) using *SQMtools* [43] and integrated into phyloseq objects with *phyloseq* (*v1.54.2*) R package [44] for subsequent statistical analyses and data visualization. Tables were formatted using the *gtsummary v2.5.0* [45] and *kableExtra v1.4.0* (https://github.com/haozhu233/kableExtra, accessed on 9 August 2026) R packages.

### 4.3. Differential Functional Analysis

All statistical analyses were performed using R (*version 4.5.0*). Differential abundance analyses were performed at the level of individual KEGG Orthologues (KOs).

For treatment-related analyses, placebo was used as the reference category. Differential abundance testing was conducted using *DESeq2* (*v1.50.2*) [46], applying negative binomial generalized linear models to raw KO count data.

Orthologues were considered significantly differentially abundant when they fulfilled both an adjusted *p*-value (Benjamini-Hochberg false discovery rate correction) below 0.05 and an absolute $Log_{2}FoldChange$ greater than 1. Exceptionally, based on the research group’s experience in this type of analysis, $Log_{2}FoldChange$ values greater than 0.5 were considered significantly abundant on an exploratory basis as long as the abundance (Reads Mean) was greater than 1400.

Positive $Log_{2}FoldChange$ values indicated over-representation in the Micodigest^®^ group, depending on the comparison performed. Negative $Log_{2}FoldChange$ values indicated over-representation in the placebo groups.

Given the exploratory nature of the study, results were interpreted considering both statistical significance and biological relevance. Orthologues displaying extremely low abundance, uncertain functional annotation, or corresponding to host-derived eukaryotic genes were excluded from downstream biological interpretation but retained in the statistical analyses.

### 4.4. KEGG Pathway Mapping and Data Visualization

To facilitate biological interpretation of differentially abundant orthologues, KOs were mapped onto KEGG metabolic pathways. Pathways were selected according to the number of significant orthologues involved, biological coherence, and relevance to the study objectives.

Graphical representations of KEGG pathways were generated using *MicrobiomeStat* (https://github.com/cafferychen777/MicrobiomeStat, accessed on 13 August 2026) and complementary R packages *clusterProfiler* (*v4.18.4*) [47], *DOSE* (*v4.4.0*) [48], *pathview* (*v1.50.0*) [49], and *enrichplot* (*v1.30.5*) [47]. Within pathway visualizations, orthologues over-represented in the Micodigest^®^ group were represented according to their corresponding positive $Log_{2}FoldChange$ values, whereas orthologues over-represented in the placebo group were represented according to negative $Log_{2}FoldChange$ values.

The significance distributions of selected differentially abundant orthologues were additionally visualized using volcano plots generated in R.

### 4.5. Ethics Statement

The study was conducted in accordance with the Declaration of Helsinki and under the same ethical approval framework as the original randomized clinical trial [8]. All participants provided written informed consent before inclusion in the study.

## 5. Conclusions

In conclusion, Micodigest^®^ supplementation was associated with functional remodeling of the gut microbiome in patients undergoing CRC surgery. Beyond the taxonomic changes previously reported, supplementation was accompanied by coordinated alterations in microbial functions involved in ecological adaptation, nutrient acquisition, sulfur metabolism, carbon utilization, bacteriophage biology and horizontal genetic exchange. These findings suggest that Micodigest® supplementation was associated with changes in multiple functional components of the intestinal microbial ecosystem. However, because the study did not include a healthy control group, these changes cannot be interpreted as evidence that Micodigest^®^ alleviated or reversed CRC-associated microbiota dysbiosis or restored the microbiome toward a healthy state.

The complex composition of Micodigest^®^, which includes extracts from multiple mushroom species together with fructooligosaccharides/inulin and other nutritional compounds, provides several plausible biological contributors to the observed functional changes. However, the present study cannot determine which specific component or bioactive compound was responsible for these effects. Likewise, the metagenomic data reflect microbial functional potential rather than actual metabolic activity, and therefore do not establish whether the observed changes resulted in corresponding alterations in microbial metabolites or host–microbiome interactions.

Taken together, the integration of taxonomic, functional and clinical findings from this randomized clinical trial supports the hypothesis that Micodigest^®^ supplementation may be associated with coordinated remodeling of the intestinal microbiome, accompanied by systemic immune changes previously reported in the clinical trial, including increased circulating lymphocyte counts and reduced neutrophil-to-lymphocyte ratios. Nevertheless, these concurrent findings do not establish a causal relationship between microbiome functional changes, immune modulation, and clinical outcomes.

Overall, these findings provide novel evidence that fungal nutraceutical supplementation may influence not only gut microbiome composition but also its functional potential in patients with CRC. More broadly, they highlight the value of functional metagenomics as a complementary approach to taxonomic profiling for identifying biologically meaningful microbial signatures associated with clinical outcomes in CRC. Future multicentre studies including healthy control populations, more frequent longitudinal sampling, chemical characterization of the nutraceutical formulation, and integrated shotgun metagenomics, metatranscriptomics, metabolomics, and host immune profiling will be important to determine whether the functional changes observed here correspond to metabolic and ecological restoration, whether they can reduce disease-associated dysbiosis, and which components of Micodigest^®^ contribute to these effects.

## Figures and Tables

**Table 1:** Differentially abundant KEGG orthologues between Micodigest^®^ and placebo groups at T2. Selected for having a relevant biological function in the study group (genes of human origin or genes with uncertain functionality are excluded).

KO	RM	*L2FC*	lfcSE	*p_Adj_*	KEGG Name	KEGG Symbol
K03277	4.86	−23.40	3.05	2.45 × 10^−11^	Heptosyltransferase IV	*WaaU, RfaK*
K06157	82.60	−5.39	1.29	1.49 × 10^−2^	Gnt-II system L-idonate transporter	*IdnT*
K00098	80.51	−3.74	0.70	1.09 × 10^−4^	L-idonate 5-dehydrogenase	*IdnD*
K11074	62.01	−2.83	0.72	3.27 × 10^−2^	Putrescine transport system permease protein	*PotI, SpuH*
K12058	311.01	−2.81	0.73	3.82 × 10^−2^	Conjugal transfer mating pair stabilization protein	*TraN*
K10126	179.69	−2.65	0.67	3.27 × 10^−2^	Two-component system, NtrC family, C4-dicarboxylate transport response regulator	*DctD*
K14138	1477.57	−0.69	0.18	3.27 × 10^−2^	Acetyl-CoA synthase	*AscB*
K02048	1430.96	−0.63	0.16	3.27 × 10^−2^	Sulfate/thiosulfate transport system substrate-binding protein	*CysP*
K00194	479.22	−0.63	0.16	3.27 × 10^−2^	Acetyl-CoA decarbonylase/synthase, CODH/ACS complex subunit delta	*CdhD, AcsD*
K10119	6411.87	−0.50	0.13	4.30 × 10^−2^	Raffinose/stachyose/melibiose transport system permease protein	*MsmG*
K13001	272.18	1.63	0.36	3.46 × 10^−3^	Mannosyltransferase	*WbyK*
K17329	68.02	3.11	0.73	1.19 × 10^−2^	N,N′-diacetylchitobiose transport system substrate-binding protein	*DasA*
K19134	152.15	3.98	0.92	9.72 × 10^−3^	CRISPR-associated protein	*Csx10*
K18942	8.80	21.66	3.08	2.16 × 10^−9^	Straboviridae DNA polymerase	*Gp43*

Abbreviations: KO, KEGG orthologue; RM, Reads Mean; *L2FC*, *Log*_2_*FoldChange*; $p_{Adj}$, *p Adjust*.

## Data Availability

The datasets generated and analyzed during the current study are available in the European Nucleotide Archive (ENA) repository (accession number PRJEB123938, ERS31136930-ERS31137011). https://www.ebi.ac.uk/ena/browser/view/PRJEB123938 (accessed on 31 August 2026), Supplementary Table S3. All data generated in this study are included in the manuscript, Supplementary Tables S1–S3, and Supplementary Figure S1. Any data not available in the paper or the repository is available from the corresponding author on reasonable request.

## Supplementary Materials

The following supporting information can be downloaded at: https://www.mdpi.com/article/10.3390/ijms27198936/s1.

## References

[1] World Health Organization (2026). Colorectal Cancer.

[2] Weinberg B.A., Sackstein P.E., Yu J., Kim R.D., Sommovilla J., Amarnath S.R., Krishnamurthi S.S. (2024). Evolving standards of care in the management of localized colorectal cancer. Am. Soc. Clin. Oncol. Educ. Book.

[3] Wong C.C., Yu J. (2023). Gut microbiota in colorectal cancer development and therapy. Nat. Rev. Clin. Oncol..

[4] White M.T., Sears C.L. (2024). The microbial landscape of colorectal cancer. Nat. Rev. Microbiol..

[5] Lianos G.D., Frountzas M., Kyrochristou I.D., Sakarellos P., Tatsis V., Kyrochristou G.D., Bali C.D., Gazouli M., Mitsis M., Schizas D. (2024). What is the role of the gut microbiota in anastomotic leakage after colorectal resection? A scoping review of clinical and experimental studies. J. Clin. Med..

[6] Mizuno M., Minato K. (2024). Anti-inflammatory and immunomodulatory properties of polysaccharides in mushrooms. Curr. Opin. Biotechnol..

[7] Araújo-Rodrigues H., Sousa A.S., Relvas J.B., Tavaria F.K., Pintado M. (2024). An overview on mushroom polysaccharides: Health-promoting properties, prebiotic and gut microbiota modulation effects and structure-function correlation. Carbohydr. Polym..

[8] Regueiro C., Diez Martín A.I., Pérez S., Daviña-Núñez C., Zarraquiños S., Remedios D., Sánchez Gómez C.A., Alonso Lorenzo S., Fernández Poceiro R., de Castro Parga M.L. (2025). The Effect of Fungal Nutraceutical Supplementation on Postoperative Complications, Inflammatory Factors and Fecal Microbiota in Patients Undergoing Colorectal Cancer Surgery with Curative Intent: A Randomized, Placebo-Controlled, Double-Blind Clinical Trial. Biomedicines.

[9] Lee K.K., Park Y., Kuehn S. (2023). Robustness of microbiome function. Curr. Opin. Syst. Biol..

[10] Li L., Wang T., Ning Z., Zhang X., Butcher J., Serrana J.M., Simopoulos C.M.A., Mayne J., Stintzi A., Mack D.R. (2023). Revealing proteome-level functional redundancy in the human gut microbiome using ultra-deep metaproteomics. Nat. Commun..

[11] Hernández-Plaza A., Deng Z., Robledo-Yagüe F., Szklarczyk D., von Mering C., Bork P., Huerta-Cepas J. (2026). eggNOG v7: Phylogeny-based orthology predictions and functional annotations. Nucleic Acids Res..

[12] Kanehisa M., Furumichi M., Sato Y., Kawashima M., Ishiguro-Watanabe M. (2023). KEGG for taxonomy-based analysis of pathways and genomes. Nucleic Acids Res..

[13] Wirbel J., Pyl P.T., Kartal E., Zych K., Kashani A., Milanese A., Fleck J.S., Voigt A.Y., Palleja A., Ponnudurai R. (2019). Meta-analysis of fecal metagenomes reveals global microbial signatures that are specific for colorectal cancer. Nat. Med..

[14] Thomas A.M., Segata N. (2019). Multiple levels of the unknown in microbiome research. BMC Biol..

[15] Dion M.B., Plante P.-L., Zufferey E., Shah S.A., Corbeil J., Moineau S. (2021). Streamlining CRISPR spacer-based bacterial host predictions to decipher the viral dark matter. Nucleic Acids Res..

[16] Cheng H., Deng H., Ma D., Gao M., Zhou Z., Li H., Liu S., Teng T. (2024). Insight into the natural regulatory mechanisms and clinical applications of the CRISPR-Cas system. Heliyon.

[17] Faure G., Shmakov S.A., Yan W.X., Cheng D.R., Scott D.A., Peters J.E., Makarova K.S., Koonin E.V. (2019). CRISPR-Cas in mobile genetic elements: Counter-defence and beyond. Nat. Rev. Microbiol..

[18] Townsend E.M., Kelly L., Muscatt G., Box J.D., Hargraves N., Lilley D., Jameson E. (2021). The human gut phageome: Origins and roles in the human gut microbiome. Front. Cell. Infect. Microbiol..

[19] Hannigan G.D., Duhaime M.B., Ruffin M.T., Koumpouras C.C., Schloss P.D. (2018). Diagnostic potential and interactive dynamics of the colorectal cancer virome. mBio.

[20] Garvey M. (2024). Intestinal dysbiosis: Microbial imbalance impacts on colorectal cancer initiation, progression and disease mitigation. Biomedicines.

[21] Krämer R. (2010). Bacterial stimulus perception and signal transduction: Response to osmotic stress. Chem. Rec..

[22] Severi E., Hood D.W., Thomas G.H. (2007). Sialic acid utilization by bacterial pathogens. Microbiology.

[23] Muramatsu M.K., Winter S.E. (2024). Nutrient acquisition strategies by gut microbes. Cell Host Microbe.

[24] Foster K.R., Schluter J., Coyte K.Z., Rakoff-Nahoum S. (2017). The evolution of the host microbiome as an ecosystem on a leash. Nature.

[25] Coyte K.Z., Schluter J., Foster K.R. (2015). The ecology of the microbiome: Networks, competition, and stability. Science.

[26] Pacheco A.R., Moel M., Segrè D. (2019). Costless metabolic secretions as drivers of interspecies interactions in microbial ecosystems. Nat. Commun..

[27] Zhao J., Hu Y., Qian C., Hussain M., Liu S., Zhang A., He R., Sun P. (2023). The interaction between mushroom polysaccharides and gut microbiota and their effect on human health: A review. Biology.

[28] Virolle C., Goldlust K., Djermoun S., Bigot S., Lesterlin C. (2020). Plasmid transfer by conjugation in Gram-negative bacteria: From the cellular to the community level. Genes.

[29] Saito A., Shinya T., Miyamoto K., Yokoyama T., Kaku H., Minami E., Shibuya N., Tsujibo H., Nagata Y., Ando A. (2007). The dasABC gene cluster, adjacent to dasR, encodes a novel ABC transporter for the uptake of N, N^′^-diacetylchitobiose in Streptomyces coelicolor A3 (2). Appl. Environ. Microbiol..

[30] Gow N.A.R., Latge J.-P., Munro C.A. (2017). The fungal cell wall: Structure, biosynthesis, and function. Microbiol. Spectr..

[31] Schäffer C., Messner P. (2017). Emerging facets of prokaryotic glycosylation. FEMS Microbiol. Rev..

[32] Lebeer S., Vanderleyden J., De Keersmaecker S.C.J. (2010). Host interactions of probiotic bacterial surface molecules: Comparison with commensals and pathogens. Nat. Rev. Microbiol..

[33] Wolf P.G., Cowley E.S., Breister A., Matatov S., Lucio L., Polak P., Ridlon J.M., Gaskins H.R., Anantharaman K. (2022). Diversity and distribution of sulfur metabolic genes in the human gut microbiome and their association with colorectal cancer. Microbiome.

[34] Wang Y., Nguyen L.H., Mehta R.S., Song M., Huttenhower C., Chan A.T. (2021). Association between the sulfur microbial diet and risk of colorectal cancer. JAMA Netw. Open.

[35] Nguyen L.H., Ma W., Wang D.D., Cao Y., Mallick H., Gerbaba T.K., Lloyd-Price J., Abu-Ali G., Hall A.B., Sikavi D. (2020). Association between sulfur-metabolizing bacterial communities in stool and risk of distal colorectal cancer in men. Gastroenterology.

[36] Ragsdale S.W., Pierce E. (2008). Acetogenesis and the Wood–Ljungdahl pathway of CO_2_ fixation. Biochim. Biophys. Acta Proteins Proteom..

[37] Jahanbakhshi S., Bibi A., Hoyd R., Dravillas C., Williams N., Zhang S., Pallerla A., Suman S., Amann J., Goruganthu M. (2026). Comparative genomics of gut commensal Lachnospiraceae and their associations with cancer treatment response with immune checkpoint inhibitors. Cancer Res..

[38] Zhu G., Schmidt O., Luan L., Xue J., Fan J., Geisen S., Sun B., Jiang Y. (2022). Bacterial keystone taxa regulate carbon metabolism in the earthworm gut. Microbiol. Spectr..

[39] Forget P., Khalifa C., Defour J.-P., Latinne D., Van Pel M.-C., De Kock M. (2017). What is the normal value of the neutrophil-to-lymphocyte ratio?. BMC Res. Notes.

[40] Zahorec R. (2001). Ratio of neutrophil to lymphocyte counts—Rapid and simple parameter of systemic inflammation and stress in critically ill. Bratisl. Med. J..

[41] Shevchenko I., Serban D., Simion L., Motofei I., Cristea B.M., Dumitrescu D., Tudor C., Dascalu A.M., Serboiu C., Tribus L.C. (2025). Clinical significance of blood cell-derived inflammation markers in assessing potential early and late postoperative complications in patients with colorectal cancer: A systematic review. J. Clin. Med..

[42] Chen X., Hu Z.-P., Yang X.-X., Huang M., Gao Y., Tang W., Chan S.Y., Dai X., Ye J., Ho P.C.-L. (2006). Monitoring of immune responses to a herbal immuno-modulator in patients with advanced colorectal cancer. Int. Immunopharmacol..

[43] Puente-Sánchez F., García-García N., Tamames J. (2020). SQMtools: Automated processing and visual analysis of’omics data with R and anvi’o. BMC Bioinform..

[44] McMurdie P.J., Holmes S. (2013). phyloseq: An R package for reproducible interactive analysis and graphics of microbiome census data. PLoS ONE.

[45] Sjoberg D.D., Whiting K., Curry M., Lavery J.A., Larmarange J. (2021). Reproducible summary tables with the gtsummary package. R J..

[46] Love M.I., Huber W., Anders S. (2014). Moderated estimation of fold change and dispersion for RNA-seq data with DESeq2. Genome Biol..

[47] Wu T., Hu E., Xu S., Chen M., Guo P., Dai Z., Feng T., Zhou L., Tang W., Zhan L.I. (2021). clusterProfiler 4.0: A universal enrichment tool for interpreting omics data. Innovation.

[48] Yu G., Wang L.-G., Yan G.-R., He Q.-Y. (2014). DOSE: An R/Bioconductor package for disease ontology semantic and enrichment analysis. Bioinformatics.

[49] Luo W., Brouwer C. (2013). Pathview: An R/Bioconductor package for pathway-based data integration and visualization. Bioinformatics.

