# Supplementary material for: Beyond Taxonomy: Fungal Nutraceutical Supplementation Modifies the Functional Potential of the Gut Microbiome in Colorectal Cancer
Source: Int J Mol Sci. 2026 Oct 8;27(19):8936. doi: 10.3390/ijms27198936 (PMC13665136; doi:10.3390/ijms27198936)
Supplement: Supplementary file 1 [file ijms-27-08936-s001.zip › ijms-4590456-supplementary.pdf]

Article

Beyond Taxonomy: Fungal Nutraceutical Supplementation Modifies the Functional Potential of the Gut Microbiome in Colorectal Cancer

Nahir Rodríguez-Costas [1,2,*], Arturo Rodríguez-Blanco[3], Cristina Regueiro Expósito[4], Joaquín Cubiella [4,5], Esteban Sinde Stompel[3], Catalina Fernández-de-Ana Portela[3], Astrid Irene Diez Martín[4], Pablo Taboada-Rodríguez[6], Belén Fontán Silva[7], María Dolores Rodríguez Fernández[7], Sara Zarraquiños[5], David Remedios[5], Cristina Alejandra Sánchez Gómez[5], Sara Alonso Lorenzo[4,8], Romina Fernández Poceiro[4], María Luisa de Castro Parga[4,8], Vicent Hernández Ramírez[4,8], Sonia Pérez-Castro[1,7]*

1. Microbiology and Infectology Research Group, Galicia Sur Health Research Institute (IIS Galicia Sur), 36312 Vigo, Spain; nahir.rodriguez@iisgaliciasur.es (N.R.-C.), sonia.maria.perez.castro@sergas.es (S.P.-C.)
2. Functional Biology and Health Sciences Department, Universidade de Vigo, 36310 Vigo, Spain; nahir.rodriguez@iisgaliciasur.es (N.R.-C.)
3. Hifas da Terra S.L., 36154 Pontevedra, Spain; arturo.rodriguez@hifasdaterra.com (A.R.-B.), esteban.sinde@hifasdaterra.com (E.S.-S.), c.fdap@hifasdaterra.com (C.F.A.-P.)
4. Research Group in Gastrointestinal Oncology Disease in Ourense, 32005 Ourense, Spain; cristina.regueiro@iisgaliciasur.es (C.R.-E.), joaquin.cubiella.fernandez@sergas.es (J.C.), astrid.diez@iisgaliciasur.es (A.I.D.-M.), Sara.Alonso.Lorenzo@sergas.es (S.A.-L.), romina.fernandez.poceiro@sergas.es (R.F.-P.), maria.luisa.de.castro.parga@sergas.es (M.L.C.-P), Vicent.Hernandez.Ramirez@sergas.es (V.H.-R.)
5. Department of Gastroenterology, Hospital Universitario de Ourense, Centro de Investigación Biomédica en Red de Enfermedades Hepáticas y Digestivas (CIBEREHD), 32005 Ourense, Spain; joaquin.cubiella.fernandez@sergas.es (J.C.), sara.zarraquinos.martinez@sergas.es (S.Z.), David.Rafael.Remedios.Espino@sergas.es (D.R.), Cristina.Alejandra.Sanchez.Gomez@sergas.es (C.A.S.-G.)
6. Escuela Técnica Superior de Ingeniería (ETSE), Universidade de Santiago de Compostela, 15782, Santiago de Compostela, Spain; pablo.taboada@gmail.com (P.T.-R.)
7. Microbiology Department, Complexo Hospitalario Universitario de Vigo (CHUVI), 36312 Vigo, Spain; Belen.Fontan.Silva@sergas.es (B.F.-S.), Maria.Dolores.Rodriguez.Fernandez@sergas.es (M.D.R.-F.), sonia.maria.perez.castro@sergas.es (S.P.-C.)
8. Department of Gastroenterology, Complexo Hospitalario Universitario de Vigo, 36312 Vigo, Spain; Sara.Alonso.Lorenzo@sergas.es (S.A.-L.), maria.luisa.de.castro.parga@sergas.es (M.L.C.-P), Vicent.Hernandez.Ramirez@sergas.es (V.H.-R.)

* Correspondence: nahir.rodriguez@iisgaliciasur.es (N.R.-C.); sonia.maria.perez.castro@sergas.es (S.P.-C.)

1. Supplementary material

Table S1. Differentially abundant KEGG orthologues between Micodigest® and placebo groups at baseline (T1).

KO	RM	*L2FC*	lfcSE	p_{Adj}	KEGG Name	KEGG Symbol
K13533	131.42	−2.76	0.71	4.74E-02	Two-component system, sporulation sensor kinase E	*KinE*
K01236	185.24	−2.71	0.63	2.45E-02	Maltooligosyltrehalose trehalohydrolase	*TreZ, GlgZ*
K16922	313.20	−2.20	0.55	3.86E-02	Putative peptide zinc metalloprotease protein	*YdH*
K06970	196.17	−1.67	0.40	3.79E-02	23S rRNA (adenine1618-N6)-methyltransferase	*RlmF*

Continued on next page

KO	RM	*L2FC*	lfcSE	p_{Adj}	KEGG Name	KEGG Symbol
K00318	100.56	−1.28	0.31	3.82E-02	Proline dehydrogenase	*PRODH, FadM, PutB*
K02395	3810.39	−0.80	0.19	2,60E-02	Peptidoglycan hydrolase	*FlgJ*
K10026	2305.78	−0.67	0.17	3.99E-02	7-carboxy-7-deazaguanine synthase	*QueE*
K11105	3755.71	−0.53	0.13	3.82E-02	Potassium/hydrogen antiporter	*CvrA, NhaP2*
K01906	79.39	1.32	0.33	3.82E-02	6-carboxyhexanoate–CoA ligase	*BioW*
K09835	101.60	3.27	0.80	3.82E-02	Prolycopene isomerase	*CrtISO, CrtH*
K18892	56.05	4.10	0.87	5.11E-03	ATP-binding cassette, sub-family B, multidrug efflux pump	*PatB, RscB, LmrC, SatB*
K18891	48.85	5.23	1.06	1.95E-03	ATP-binding cassette, sub-family B, multidrug efflux pump	*PatA, RscA, LmrC, SatA*
K22394	6.71	21.91	2.86	6.05E-11	Flavin reductase (NADH) subunit 2	*Nfr2*
K18795	10.49	22.54	2.24	6.36E-20	Beta-lactamase class A *CARB-1*, PSE family	*BlaCARB-1*

Abbreviations: KO, KEGG orthologue; RM, Reads Mean; *L2FC*, $Log_2FoldChange$; p_{Adj}, *p Adjust*

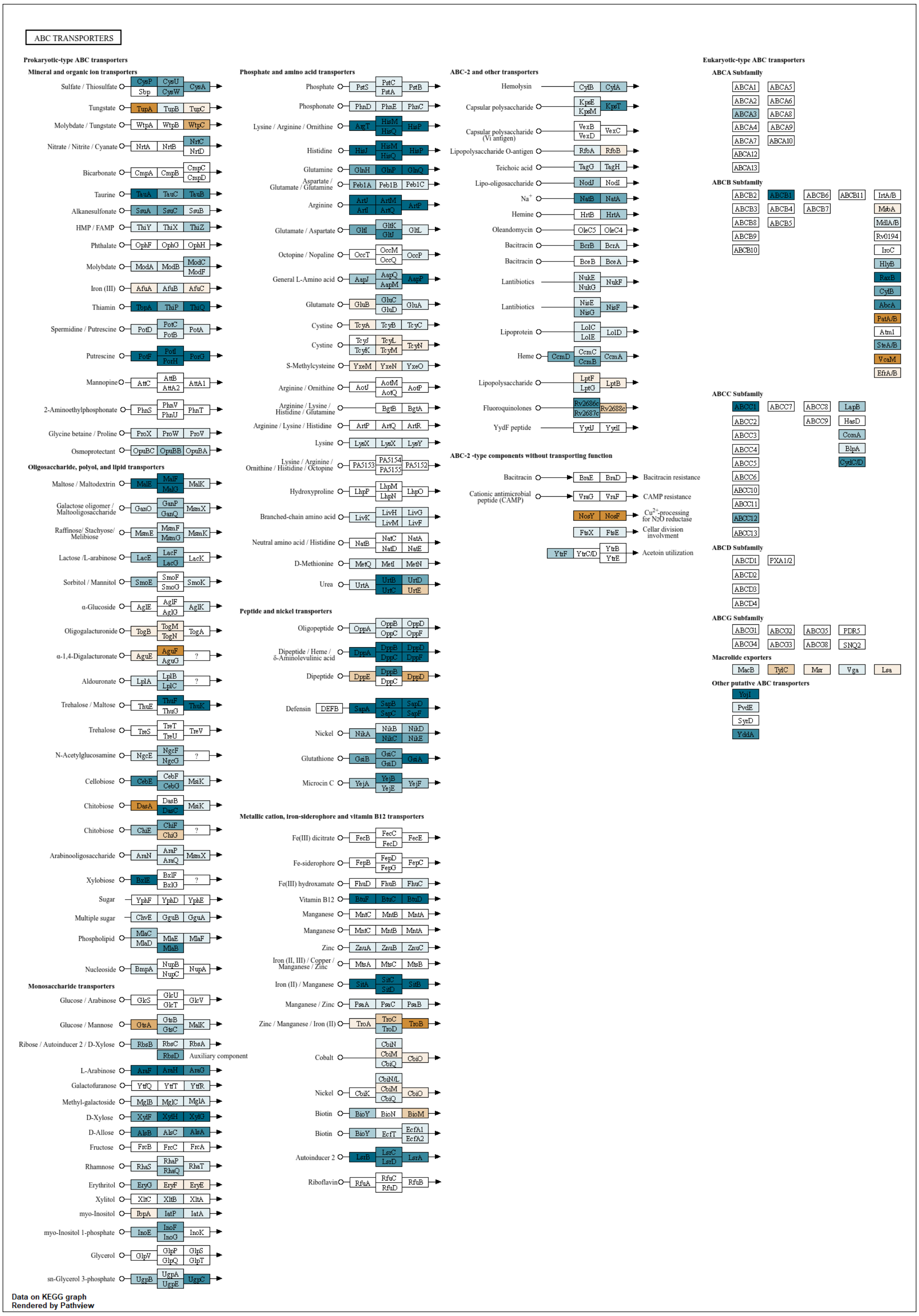

Figure S1. ABC transporters pathway (map02010). Positive $Log_2FoldChange$ values (orange) correspond to over-represented orthologs in Micodigest® at T2, and negative values (blue) to under-represented orthologs in Micodigest® at T2. Enzymes significantly under-represented in Micodigest® (*PotI*, *CysP* and *MsmG*) are shown in blue (p_{Adj} = 3.27 × 10^{-2}, p_{Adj} = 3.27 × 10^{-2}, p_{Adj} = 4.30 × 10^{-2}, respectively). Enzyme significantly over-represented in Micodigest® (*DasA*) is shown in orange (p_{Adj} = 1.19 × 10^{-2}).

Table S2. Differentially abundant KEGG orthologues between Micodigest® and placebo groups at T2 in Conventional Surgery subgroup.

KO	RM	*L2FC*	lfcSE	p_{Adj}	KEGG Name	KEGG Symbol
K00098	68.93	−4.02	0.98	2.92E-02	L-idonate 5-dehydrogenase	*IdnD*
K20609	64.40	−3.42	0.81	2.91E-02	Putative aminopeptidase	*SgcX*
K20483	161.39	−2.87	0.69	2,92E-02	Class I lanthipeptide synthase	*LanB, NisB, SpaB, EpiB*
K03608	579.47	−0.58	0.14	3.21E-02	Cell division topological specificity factor	*MinE*
K10909	1055.38	1.89	0.48	4.66E-02	Two-component system, autoinducer 2 sensor kinase/phosphatase	*LuxQ*
K16267	39.12	3.32	0.85	4.87E-02	Zinc and cadmium transporter	*ZipB*
K14387	132.19	3.91	0.85	5.75E-03	Solute carrier family 5, member 7	*SLC5A7, CHT1*
K19342	64.90	4.61	0.91	7.53E-04	Copper chaperone	*NosL*
K21252	593.09	5.92	1.41	2.92E-02	Fosfomycin resistance protein	*FosX*
K07516	20.06	7.24	1.76	2.92E-02	3-hydroxyacyl-CoA dehydrogenase	*FadN*
K06953	28.02	7.72	1.88	2.92E-02	Uncharacterized protein	*NA*
K17229	56.11	8.73	1.65	3.38E-04	Sulfide dehydrogenase [flavocytochrome c] flavoprotein chain	*FccB*
K19952	13.48	21.12	3.07	2.48E-08	Guanine nucleotide exchange factor	*RABIF, MSS4, DSS4*
K05953	17.87	21.51	2.85	3.18E-10	Nicotianamine synthase	*NAS*

Abbreviations: KO, KEGG orthologue; RM, Reads Mean; *L2FC*, *$Log_2FoldChange$*; p_{Adj}, *p Adjust*

Table S3. Summary of sample metadata including study treatment (Micodigest or Placebo), time (Before treatment (T1) or Post treatment (T2)), and corresponding European Nucleotide Archive (ENA) accession IDs for each sample.

Treatment	Time	ENA ID
Micodigest®	2	ERS31136930
Micodigest®	1	ERS31136931
Micodigest®	1	ERS31136932
Placebo	1	ERS31136933
Micodigest®	1	ERS31136934
Micodigest®	1	ERS31136935
Placebo	1	ERS31136936
Micodigest®	2	ERS31136937
Placebo	2	ERS31136938
Placebo	1	ERS31136939
Micodigest®	2	ERS31136940
Micodigest®	1	ERS31136941
Micodigest®	2	ERS31136942
Placebo	1	ERS31136943
Micodigest®	2	ERS31136944
Placebo	2	ERS31136945
Micodigest®	1	ERS31136946
Micodigest®	2	ERS31136947
Micodigest®	1	ERS31136948
Placebo	2	ERS31136949
Micodigest®	2	ERS31136950
Micodigest®	1	ERS31136951
Micodigest®	2	ERS31136952
Micodigest®	1	ERS31136953
Micodigest®	1	ERS31136954
Micodigest®	1	ERS31136955
Micodigest®	1	ERS31136956
Micodigest®	2	ERS31136957
Micodigest®	1	ERS31136958
Micodigest®	2	ERS31136959
Micodigest®	2	ERS31136960

Continued on next page

Treatment	Time	ENA ID
Micodigest®	2	ERS31136961
Micodigest®	1	ERS31136962
Placebo	1	ERS31136963
Placebo	1	ERS31136964
Placebo	1	ERS31136965
Micodigest®	2	ERS31136966
Placebo	2	ERS31136967
Placebo	1	ERS31136968
Placebo	1	ERS31136969
Placebo	2	ERS31136970
Placebo	2	ERS31136971
Micodigest®	2	ERS31136972
Micodigest®	1	ERS31136973
Micodigest®	1	ERS31136974
Micodigest®	2	ERS31136975
Micodigest®	1	ERS31136976
Micodigest®	2	ERS31136977
Placebo	1	ERS31136978
Placebo	1	ERS31136979
Micodigest®	2	ERS31136980
Placebo	2	ERS31136981
Placebo	2	ERS31136982
Micodigest®	1	ERS31136983
Placebo	1	ERS31136984
Placebo	1	ERS31136985
Micodigest®	1	ERS31136986
Micodigest®	1	ERS31136987
Micodigest®	2	ERS31136988
Micodigest®	1	ERS31136989
Micodigest®	2	ERS31136990
Placebo	2	ERS31136991
Placebo	1	ERS31136992
Micodigest®	2	ERS31136993
Placebo	1	ERS31136994
Placebo	2	ERS31136995
Micodigest®	1	ERS31136996
Micodigest®	1	ERS31136997
Placebo	1	ERS31136998
Micodigest®	2	ERS31136999
Micodigest®	2	ERS31137000
Placebo	1	ERS31137001
Placebo	2	ERS31137002
Placebo	2	ERS31137003
Micodigest®	1	ERS31137004
Micodigest®	1	ERS31137005
Micodigest®	1	ERS31137006
Placebo	1	ERS31137007
Micodigest®	2	ERS31137008
Micodigest®	2	ERS31137009
Placebo	2	ERS31137010
Placebo	1	ERS31137011
